# Terahertz Dipole Nanoantenna Arrays: Resonance Characteristics

**DOI:** 10.1007/s11468-012-9439-0

**Published:** 2012-08-24

**Authors:** Luca Razzari, Andrea Toma, Matteo Clerici, Mostafa Shalaby, Gobind Das, Carlo Liberale, Manohar Chirumamilla, Remo Proietti Zaccaria, Francesco De Angelis, Marco Peccianti, Roberto Morandotti, Enzo Di Fabrizio

**Affiliations:** 1 Fondazione Istituto Italiano di Tecnologia, Via Morego 30, 16163 Genoa, Italy; 2 INRS-EMT, 1650 Boulevard Lionel Boulet, Varennes, Québec Canada J3X 1S2

**Keywords:** Terahertz spectroscopy, Nanostructure fabrication, Plasmonics, Nanophotonics

## Abstract

Resonant dipole nanoantennas promise to considerably improve the capabilities of terahertz spectroscopy, offering the possibility of increasing its sensitivity through local field enhancement, while in principle allowing unprecedented spatial resolutions, well below the diffraction limit. Here, we investigate the resonance properties of ordered arrays of terahertz dipole nanoantennas, both experimentally and through numerical simulations. We demonstrate the tunability of this type of structures, in a range (∼1–2 THz) that is particularly interesting and accessible by means of standard zinc telluride sources. We additionally study the near-field resonance properties of the arrays, finding that the resonance shift observed between near-field and far-field spectra is predominantly ascribable to ohmic damping.

## Introduction

In the last years, optical nanoantennas [1, 2], with their ability to focus light well beyond the diffraction limit, have shown to offer new exciting opportunities for photonics and optical spectroscopy. For example, the strong and highly localized field associated with these structures has been used to significantly enhance high-harmonic generation [3], fluorescence [4, 5], Raman scattering [6, 7], and direct infrared absorption [8, 9].

In the terahertz spectral region, nanoslot antennas (i.e., nanorectangular apertures on a thin metal layer) have first been proposed [10–14]. These structures exhibit very high field enhancement, but present limitations when compared to metallic rod nanoantennas, e.g., they do not localize the radiation along the length of the slot. In a very recent work, we have shown that terahertz resonant dipole nanoantennas can succeed in combining strong localization and high field enhancement [15], opening interesting perspectives for applying the nanoantenna concept in terahertz science and technology.

In this work, we investigate the properties of ordered arrays of terahertz dipole nanoantennas. We experimentally demonstrate the tunability of their resonance characteristic by varying the nanoantenna length and perform a direct comparison with numerical electromagnetic simulations and a Fabry–Perot-like analytical model. Terahertz dipole nanoantenna arrays combine a strongly localized field enhancement with a spatially extended interaction with the incoming radiation, which improves far-field detectability, and can thus find important applications as a platform for ultra-sensitive terahertz spectroscopy and antenna-enhanced terahertz nonlinear experiments.

## Experimental

**Fig. 1:**
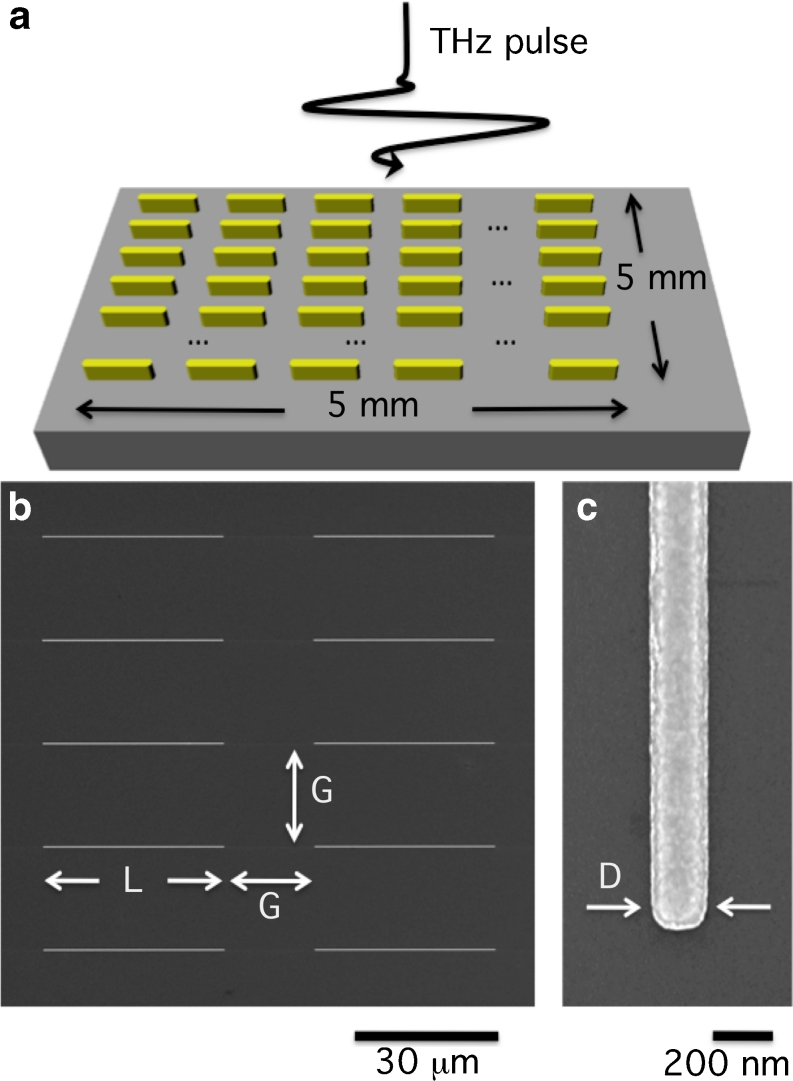
**a** Sketch of the fabricated terahertz nanoantenna arrays. **b** SEM image of a detail of the array with *L* = 40 μm. **c** Magnification of the nanoantenna tip. (*L* nanoantenna length; *D* nanoantenna width; *G* array spacing)

To fabricate the terahertz nanoantenna arrays, we employed the following procedure: a 120-nm thick poly(methylmethacrylate) (PMMA) layer was spin-coated on a 500-μm thick, high-resistivity (>10 kΩ cm) (100)-oriented silicon substrate. To prevent charging effects during the electron exposure, a 10-nm thick Al layer was thermally evaporated on the PMMA surface. Electron beam direct-writing of the nanoantenna patterns was carried out using a high-resolution Raith150-Two e-beam writer at 15 keV beam energy and 520 μC/cm^2^ exposure dose. After the Al removal in a KOH solution, the exposed resist was developed in a conventional solution of MIBK/isopropanol (IPA) (1:3) for 30 s. Then, electron beam evaporation was employed to produce a 5-nm adhesion layer of titanium with a 0.3-Å/s deposition rate in an HV chamber (base pressure 10^−7^ mbar). In situ thermal evaporation of 60 nm gold film with a 0.3-Å/s deposition rate was accomplished by means of a high temperature source mounted inside the vacuum chamber. After the film deposition, the unexposed resist was removed with acetone and rinsed out in IPA. O_2_ plasma ashing was used to remove residual PMMA resist and organic contaminants for an improved lift-off. The fabricated two-dimensional arrays cover an area of 5 × 5 mm^2^ and are composed of aligned gold nanoantennas with a fixed spacing *G* = 20 μm in both directions on the plane (Fig. 1). In order to investigate the possibility of tuning the operating resonance of terahertz nanoantenna arrays, we have prepared a set of five samples with antennas of different lengths (*L* = 30, 35, 40, 50, and 60 μm, respectively) and fixed width (*D* = 200 nm) and height (60 nm).

The nanoantenna array characterization at terahertz frequencies was performed exploiting a time-domain spectroscopy setup [16]. The terahertz beam was generated by optical rectification in a 500-μm thick (110)-ZnTe crystal, using 800 nm pulses (130 fs time duration, 1.6 mJ energy) delivered by a commercial Ti/sapphire source (Spitfire Pro, Spectra-Physics). A black polyethylene pellicle, transparent to terahertz radiation, was employed to block the remaining 800 nm light transmitted through the ZnTe crystal. The nanoantenna array was illuminated by a collimated terahertz beam (7 mm beam diameter) and the transmitted beam was then focused on a second 500-μm thick (110)-ZnTe crystal, which served as a detector in a classical electro-optical sampling arrangement [17]. All the measurements were performed in a nitrogen-purged environment.

## Results and Discussion

**Fig. 2:**
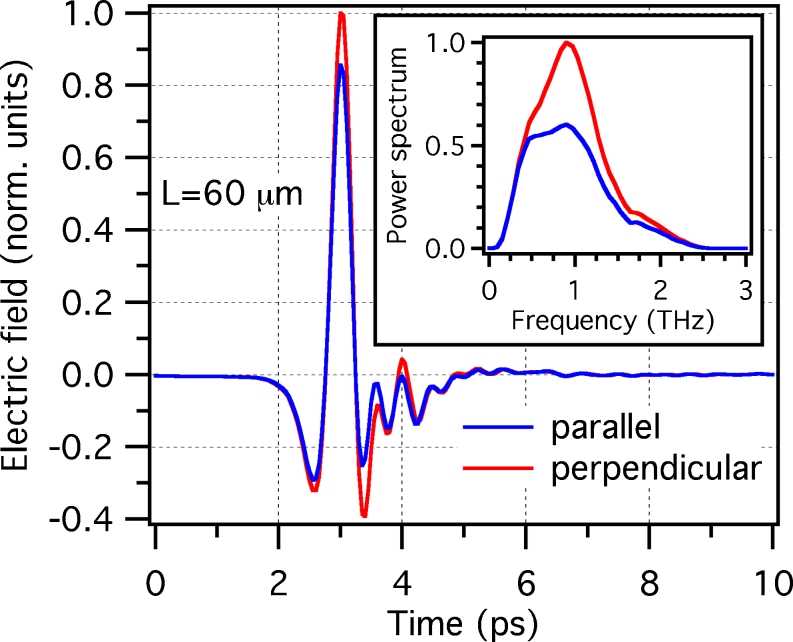
**a** Terahertz temporal waveforms transmitted through the sample, in case of polarization set parallel (*blue curve*) and perpendicular (*red curve*) to the long axis of the nanoantennas. (*Inset*: power spectra of the transmitted terahertz pulses for the two polarization states)

**Fig. 3:**
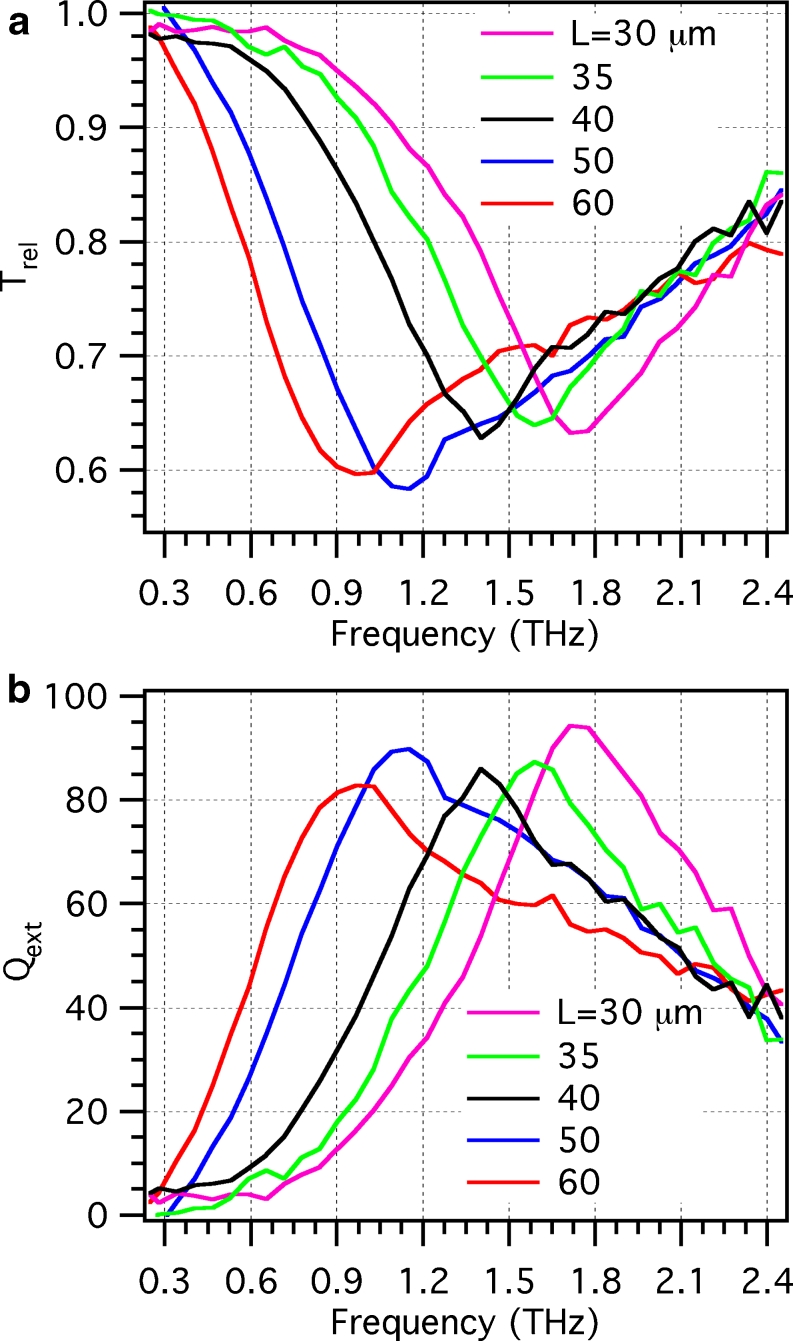
**a** Relative transmission *T*_rel_ of the nanoantenna arrays as a function of frequency, measured for the five samples with different nanoantenna lengths. **b** Extinction efficiency *Q*_ext_ as a function of frequency, evaluated from the experimental data using Eq. 1 (colors as in **a**)

The fabricated samples were characterized using far-field extinction spectroscopy [18]. We measured the THz pulses transmitted through the arrays (normal incidence), for the two cases of polarization set parallel and perpendicular to the long axis of the nanoantennas (see Fig. 2 as an example, for the case of *L* = 60 μm). Since the nanoantenna-covering factor (ratio of the area covered by the nanoantennas divided by the overall illuminated area) is less than 0.5 %, the array transmission in the case of polarization set along the short axis of the nanoantennas is found to be substantially identical to the one of a reference silicon substrate with no nanoantennas. This allows us to extract the resonance properties of the arrays by simply dividing the power spectrum of the transmitted pulse for long axis excitation by the one taken in the case of short axis excitation. The quantity thus obtained is named *relative transmittance* (*T*_rel_) and is reported in Fig. 3a, for the entire set of samples. One can notice that transmission is significantly reduced, down to values close to 60 %, in conjunction with a broad resonance behavior, whose dip redshifts when the length of the nanoantennas composing the array is increased.

From *T*_rel_, we can then estimate the nanoantenna extinction efficiency *Q*_ext_ as:

1 $$ {Q_{\text{ext}}} = \frac{{{\sigma_{\text{ext}}}}}{{{\sigma_{\text{geo}}}}} = \frac{{A\left( {1 - {T_{\text{rel}}}} \right)}}{\text{NLD}} $$

where *σ*_ext_ is the nanoantenna extinction cross section, *σ*_geo_ = LD the geometric cross section, *A* the illuminated area, while *N*, *L*, and *D* are the number, length, and width of the illuminated nanoantennas, respectively. Figure 3b evidences that, for all the samples under investigation, the peak value of the extinction efficiency is around 90, meaning that, under resonance conditions, the dipole nanoantennas increase their effective cross section of about 90 times.

To better understand the resonance characteristics of terahertz nanoantenna arrays, we performed numerical simulations using a finite integration technique-based commercial software [19]. We considered gold nanoantennas with a rectangular lateral section of 200 × 60 nm^2^, capped with hemicylindrical terminations of radius *R* = 100 nm. The nanoantenna sharp edges were blended with a curvature radius of 20 nm, in order to better resemble the fabricated structures. The nanoantennas were supposed to be entirely embedded in a background medium with dielectric constant ${\varepsilon_{\text{bg}}} = {{{\left( {1 + n_{\text{s}}^2} \right)}} \left/ {2} \right.}$ [20], where *n*_s_ = 3.42 is the refractive index of the silicon substrate [21]. Regarding the dielectric constant of gold, we considered the values reported in [22]. Simulations were all performed under plane wave illumination at normal incidence, with polarization set along the long axis of the nanoantennas.

To numerically evaluate *Q*_ext_ and thus extract the nanoantenna resonance properties, we calculated the total absorption cross section *σ*_abs_ and total scattering cross section *σ*_sca_ and then used the simple relation: ${Q_{\text{ext}}} = \frac{{{\sigma_{\text{ext}}}}}{{{\sigma_{\text{geo}}}}} = {Q_{\text{abs}}} + {Q_{\text{sca}}} = \frac{{{\sigma_{\text{abs}}}}}{{{\sigma_{\text{geo}}}}} + \frac{{{\sigma_{\text{sca}}}}}{{{\sigma_{\text{geo}}}}}$, where *Q*_abs_ and *Q*_sca_ are the absorption and scattering efficiencies, respectively.

**Fig. 4:**
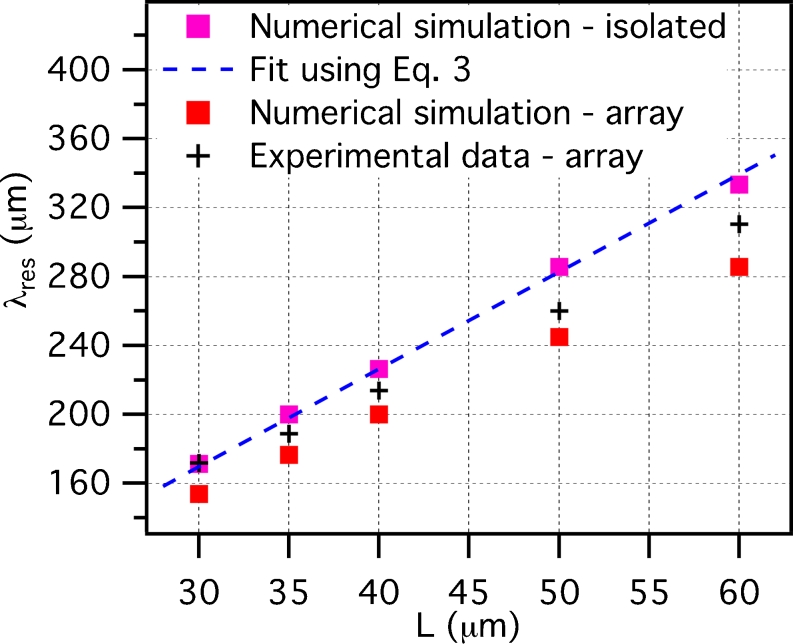
Resonance wavelength *λ*_res_ as a function of the nanoantenna length *L*. *Magenta squares*: numerical simulation for an isolated nanoantenna; *dashed blue line*: best fit of the simulation regarding the isolated nanoantenna case, using Eq. 3; *red squares*: numerical simulations using periodic boundary conditions (20 μm spacing in both directions on the plane of the array); *black crosses*: experimental data extracted from Fig. 3

At first, we studied the case of a single isolated nanoantenna. Figure 4 (magenta squares) represents the numerical result of the resonance wavelength *λ*_res_ for *L* = 30, 35, 40, 50, and 60 μm. As can be seen, by increasing the antenna length, one finds an almost perfectly linear increase of the resonance wavelength. The antenna resonance can even be estimated analytically using a simple Fabry–Perot-like model, which considers the antenna as the equivalent of a cavity for the surface charge wave [23, 24]. In this framework, the lowest order (dipolar) resonance wavelength *λ*_res_ can be written as:

2 $$ \frac{{{\lambda_{\text{res}}}}}{{2{n_{\text{eff}}}}} = L + 2\delta, $$

where *n*_eff_ is the effective index of the surface charge wave and *δ* is introduced to take into account the apparent increase of the antenna length, due to the reactance of the antenna ends [23]. Usually, *δ* is of the order of the lateral dimension of the antenna [23, 24] and can thus be neglected in our case, since *L* ≫ *D*. Equation 2 can then be simplified as follows:

3 $$ {\lambda_{\text{res}}} = 2{n_{\text{eff}}}L. $$

We can now use Eq. 3 to fit the results of the simulation (dashed blue curve in Fig. 4), obtaining a value for the effective index: *n*_eff_ = 2.83. It is interesting to note that ${{n}_{{{\text{eff}}}}} > {{n}_{{{\text{bg}}}}} = \sqrt {{{{\varepsilon }_{{{\text{bg}}}}}}}  = 2.52$. This suggests that a gold nanowire with lateral section as in the studied case (200 × 60 nm^2^) cannot be considered as an ideal conductor at terahertz frequencies and thus shows an effective index for the surface charge wave larger than the background index.

If we now try to compare the just-reported simulations for isolated nanoantennas with the experimental resonance wavelengths that can be extracted from Fig. 3 (black crosses in Fig. 4), we notice that the experimental values present a shift towards shorter wavelengths. We attribute this shift to the long-range dipolar interaction between neighboring nanoantennas in the closely spaced array, which is known to affect the resonance properties of nanoantenna arrays [9]. In practice, in an array, each nanoantenna responds not only to the incident electromagnetic field, but also to the field created by the other induced dipoles.

To prove this, we have repeated the numerical simulations, considering this time periodic boundary conditions, to accurately simulate the response of an array with a spacing of 20 μm in both directions on the plane. The results thus obtained (red squares in Fig. 4) show a blueshift of the array resonance frequency, confirming the origin of the shift observed in the experimental data. It can be seen that the numerically estimated shift is somewhat larger than the one found experimentally. This difference may be due to the uncertainty in the effective dielectric constants of the materials involved [25].

**Fig. 5:**
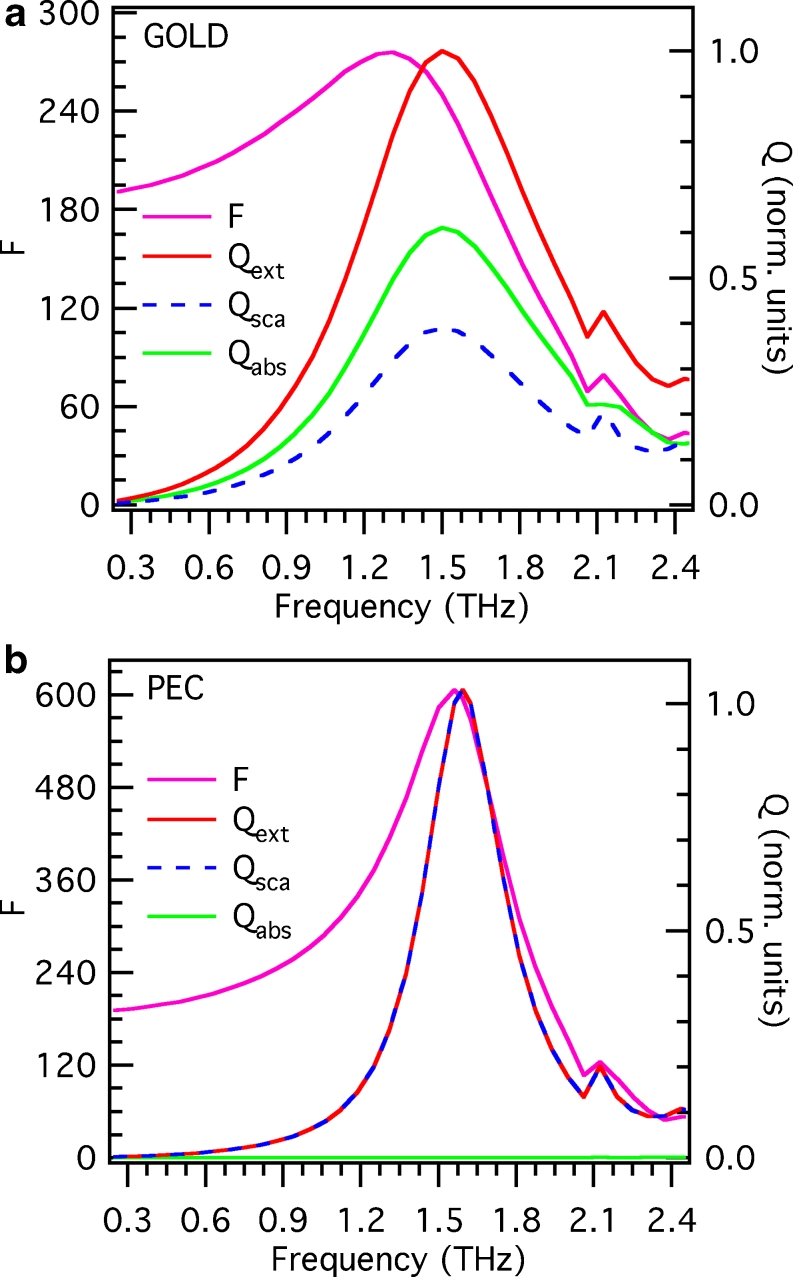
**a** Comparison between simulated near-field and far-field resonance spectra, for the array of gold nanoantennas with length *L* = 40 μm (dielectric constant of gold taken from [22]). *Left vertical axis*: field enhancement factor *F* (*magenta line*) as a function of frequency (*F* is evaluated at the nanoantenna end, on a plane that is perpendicular to the propagation direction of the illuminating wave and cuts the nanoantenna at its half height). *Right vertical axis*: normalized values of the extinction, scattering, and absorption efficiency *Q*_ext_, *Q*_sca_, and *Q*_abs_ (*red*, *dashed blue*, and *green line*, respectively) as a function of frequency. **b** Same as in **a**, substituting gold with a perfect electric conductor

For future applications, exploiting the field enhancement in close proximity of terahertz nanoantennas, it is interesting to study the near-field resonance properties of these structures and compare them with the above reported far-field resonance characteristics. For the sake of simplicity, let us consider only the case of the nanoantenna array with *L* = 40 μm. Figure 5a (magenta curve) shows the field enhancement factor *F* (defined as the ratio of the local electric field to the free-space field) at the nanoantenna extremity as a function of frequency. A broad resonance behavior can be observed, peaked at around 1.3 THz, showing values of the field enhancement of the order of few hundreds. On the same graph (right vertical axis), the values of *Q*_abs_, *Q*_sca_, and their sum *Q*_ext_ are reported. Interestingly, we find that both absorption and scattering significantly contribute to the far-field resonance properties of the nanoantennas, showing a peak at the same frequency of 1.5 THz. The near-field resonance peak is thus found to be redshifted of around 200 GHz with respect to the far-field peak. This kind of behavior has been already observed in nanoantennas at optical frequencies and has been attributed to plasmon damping [26–28]. In practice, the nanoantenna can be seen as a driven and damped harmonic oscillator. While the energy dissipation of the oscillator can be associated to the far-field extinction cross section, the oscillator amplitude is a measure of the near-field amplitude. When damping is present, the oscillator amplitude peaks at a frequency lower than the natural frequency of the oscillator, while the maximum of the energy dissipation remains unshifted [28]. The magnitude of the shift is directly related to the total damping of the system, given as the sum of the intrinsic (ohmic) damping within the metal and the radiative damping [26]. To get a better insight into the nature of this phenomenon at terahertz frequencies, we have substituted in the simulation a perfect electric conductor (PEC, i.e., a material with infinite conductivity) in place of the previously considered gold with realistic dielectric constant. Figure 5b shows the results of this procedure. As expected, in the case of a PEC nanoantenna, the contribution of absorption vanishes and the far-field properties are ruled by scattering. In addition to a considerably sharper resonance, we observe that the near-field resonance peak is almost coincident with the far-field peak. We can thus conclude that the significant near-field resonance shift observed in terahertz nanoantennas is mainly a consequence of ohmic damping.

## Conclusion

We have studied, both experimentally and numerically, the resonance characteristics of terahertz nanoantenna arrays. We have shown that their resonance peak can be tuned by varying the length of the nanoantennas, covering a significant portion of the band offered by standard ZnTe-based terahertz sources. The experimental results are found to be in fair agreement with electromagnetic simulations. In addition, we have found that the near-field resonance wavelength is redshifted when compared to the far-field peak, due to the ohmic damping within the metal constituting the nanoantennas.

Terahertz dipole nanoantenna arrays can be ideal substrates for terahertz few-molecule spectroscopy. Moreover, the exploitation of their characteristic field enhancement may lead to the practical implementation of localized nonlinear experiments at terahertz frequencies.
